# Mechanistic studies on *δ*-aminolevulinic acid uptake and efflux in a mammary adenocarcinoma cell line

**DOI:** 10.1038/sj.bjc.6601066

**Published:** 2003-07-01

**Authors:** S Correa García, A Casas, C Perotti, A Batlle, M Bermúdez Moretti

**Affiliations:** 1 Centro de Investigaciones sobre Porfirinas y Porfirias (CIPYP), FCEN, University of Buenos Aires, Argentina; CONICET, Ciudad Universitaria, Pabellón II, 2° Piso, Buenos Aires, 1428 Argentina

**Keywords:** *δ*-aminolevulinic acid, photodynamic therapy, ALA uptake, ALA efflux

## Abstract

*δ*-aminolevulinic acid (ALA) is the precursor in the biosynthesis of porphyrins. The knowledge of both the regulation of ALA entrance and efflux from the cells and the control of porphyrin biosynthesis is essential to improve ALA-mediated photodynamic therapy. In this work, we studied the regulation of ALA uptake and efflux by endogenously accumulated ALA and/or porphyrins in murine mammary adenocarcinoma cells. Under our set of conditions, the haem synthesis inhibitor succinyl acetone completely prevented porphobilinogen and porphyrin synthesis from ALA, and led to an increase in the intracellular ALA pool. However, neither intracellular ALA nor porphyrin pools regulate ALA uptake or efflux during the first 15 min of the process. Based on temperature dependence data, ALA but not *γ*-aminobutyric acid (GABA) efflux is mediated by a diffusion mechanism. Moreover, the addition of extracellular GABA not only did not influence the rate of ALA efflux but on the contrary it affected ALA uptake, showing the contribution of a saturable mechanism for the uptake, but not for the efflux of ALA from the cells.

*δ*-aminolevulinic acid (ALA) is the precursor in the biosynthesis of porphyrins, which in association with several proteins are essential for the utilisation and metabolism of oxygen. The first step of the porphyrin biosynthetic pathway is the synthesis of ALA by a condensation reaction between succinyl CoA and glycine, catalysed by the enzyme ALA synthase. This enzyme is rate limiting, tightly regulated by feedback inhibition (Rimington, 1966). The second rate limiting step in haem synthesis is the incorporation of ferrous iron into protoporphyrin IX (PPIX), a reaction catalysed by the enzyme ferrochelatase. Owing to this slow conversion of PPIX to haem (Pottier *et al*, 1986) when exogenous ALA is administered, high tissue levels of porphyrin intermediates are found (Malik and Djaldetti, 1979; Sinna *et al*, 1981).

Photodynamic therapy (PDT) is a nonthermal technique for inducing tissue damage with light following administration of a light-activated photosensitising drug that can be selectively retained in malignant or diseased lesions relative to normal adjacent tissue (Dougherty *et al*, 1978). In addition, the fluorescence of photosensitising chromophores has been exploited for the visualisation and diagnosis of early-stage superficial cancers (Kriegmair *et al*, 1996). It has been demonstrated that sufficient PPIX, a potent photosensitiser, is synthesised by exogenous ALA administration to produce a photodynamic effect following exposure to light (Kennedy *et al*, 1990; Fukuda *et al*, 1993a).

ALA-induced PPIX accumulation has been shown to be preferentially greater in certain tumoral cells (Navone *et al*, 1988), primarily due to the reduced activity of ferroquelatase and a relative enhancement of porphobilinogen (PBG) deaminase activity in these cells (Navone *et al*, 1991). The success of ALA-mediated photosensitisation will depend on an efficient ALA uptake, a low ALA efflux and an efficient conversion of ALA into porphyrins.

Several reports regarding ALA uptake systems have appeared. Thus, some authors demonstrated that ALA is taken up through the di- and tripeptide transporters PEPT1 and PEPT2 (Döring *et al*, 1998; Novotny *et al*, 2000; Whitaker *et al*, 2000). Other authors reported that BETA transporters are involved in ALA transport (Rud *et al*, 2000). The BETA transporter family comprise GAT-1 to GAT-3, BGT-1 and TAUT transport systems (Palacín *et al*, 1998). Using murine mammary adenocarcinoma cultured cells, we have recently demonstrated that ALA is incorporated by two different processes (Bermúdez Moretti *et al*, 2002). One of these processes is passive diffusion that is significant at shorter incubation intervals. The other is an active transport system that becomes very important after the first 15 min of incubation. The latter system is mediated by one of the BETA transporters, very likely GAT-2 (Bermúdez Moretti *et al*, 2002).

The knowledge of both the regulation of ALA entrance into the cells and the control of porphyrin biosynthesis is essential to improve ALA-mediated PDT. In this work, we studied the regulation of ALA uptake by endogenously accumulated ALA and/or porphyrins in murine mammary adenocarcinoma cells. We have also determined the nature of the ALA efflux process.

## MATERIALS AND METHODS

### Cell line and cell culture

Cell line LM3 (Werbajh *et al*, 1998) derived from the murine mammary adenocarcinoma M3 was cultured in minimum essential Eagle's medium, supplemented with 2 mM L-glutamine, 40 *μ*g gentamycin ml^−1^ and 5% fetal bovine serum, and incubated at 37°C in an atmosphere containing 5% CO_2_. A total of 3.5 × 10^4^ cells well^−1^ were seeded into 24-well plates and the medium was renewed 24 h before the experiment.

### Chemicals

[4-^14^C]ALA hydrochloride and [^14^C(U)]*γ*-aminobutyric acid (GABA) were obtained from New England Nuclear, ALA, GABA, succinyl acetone (SA) and metabolic inhibitors were obtained from Sigma Chemical Co., St Louis, USA. Other chemicals were of analytical grade.

### ALA and GABA preparation

Unlabelled ALA or GABA were dissolved in phosphate-buffered saline (PBS) and pH was adjusted to 7.4 with NaOH. [^14^C]ALA and [^14^C]GABA were added so that the final solution contained 0.0222 and 0.0111 MBq ml^−1^, respectively.

### Uptake measurements

Uptake measurements were performed 72 h after seeding, when the cells were nearly confluent. The cells were washed twice with 0.5 ml PBS–0.1% glucose preheated at 37°C and incubated with 0.3 ml radiolabelled 0.6 mM ALA or GABA prepared in PBS–0.1% glucose at 37°C. At the indicated times, the reaction was stopped by washing the cells four times with 0.5 ml ice-cold PBS containing either 1 mM ALA or 1 mM GABA to remove nonspecific binding. Then the cells were disrupted in 0.1 mM NaOH and transferred to vials containing scintillation fluid (OptiPhase-Hisafe 3, Perkin-Elmer, England). The radioactive content of the samples was determined.

### Efflux experiments

The cells were loaded with 0.6 mM ^14^C-ALA or ^14^C-GABA for 15 min, washed four times with PBS containing either 1 mM ALA or 1 mM GABA to remove nonspecific binding and further incubated with PBS at 37°C. Radioactivity within the cells and in the medium was measured at different times of incubation, after adding 0.1 mM NaOH.

### ALA and PBG determinations

The cells were seeded in 100 mm dishes. After 72 h, the medium was removed and the cells were exposed for 3 h to 0.6 mM ALA in a medium without serum. Afterwards, the cells were washed four times with PBS and 5% TCA was added. After scrapping, the cells were centrifuged and the supernatant was employed for ALA and PBG determinations. Modifications of the Mauzerall and Granick (1956) method were used. Briefly, for ALA determination, a condensation reaction was developed in the presence of acetyl acetone and the resulting pyrroles were quantified by addition of the Ehrlich reactive. For PBG determination, the Ehrlich reactive was added to the deproteinised TCA supernatant. ALA values were obtained by subtracting PBG values to the total condensed pyrroles.

### Porphyrin synthesis

Porphyrins accumulated within the cells were extracted twice with 5% HCl, leaving the cells standing for 30 min in the presence of the acid at 37°C. For media determinations, 5% HCl was added and measured directly. These conditions proved to be optimal for total PPIX extraction. The excitation and emission wavelengths of light used producing the highest fluorescence were 406 and 604 nm, respectively. PPIX (Porphyrin Products, Logan, UT, USA) was used as a reference standard. All the experiments were performed at 0.6 mM ALA, because porphyrin synthesis is saturated at this concentration.

### Cell number

The number of cells seeded per well and employed for the calculations were determined by counting viable cells with the Trypan blue-exclusion method.

### Statistic analysis

Quadruplicates were run for each point in every experiment and the values presented are the average of three experiments (mean±s.d.). The deviation of these values from the mean was less than 15%. A paired Student's *t*-test was used to determine statistical significance between means. *P* values <0.05 were considered significant.

## RESULTS

Cells were incubated with 0.6 mM ALA in the presence or absence of 0.5 mM SA for 3 h, then ALA, PBG and porphyrins were measured (Table 1

**Table 1:** Intracellular pools of ALA, PBG and porphyrins

	**−ALA−SA**	**+ALA−SA**	**+ALA+SA**
ALA (pmol 10^−5^ cell)	ND	13.05±0.90	105.4±7.1
PBG (pmol 10^−5^ cell)	ND	15.12±1.10	ND
Porphyrins(pmol 10^−5^ cell)	0.56±0.02	13.01±0.70	0.38±0.02

Cells were exposed for 3 h to 0.6 mM ALA in the presence or absence of 0.5 mM SA in a medium without serum. Afterwards, ALA, PBG and porphyrins were determined according to the procedures described in Materials and Methods. −ALA+SA controls (cells in the presence of SA without exposure to ALA) have been subtracted from +ALA+SA values. ND: nondetectable by this method.

). Upon exposure to 0.5 mM SA, PBG and porphyrin biosynthesis is completely eliminated. The presence of a higher intracellular pool of ALA (105.4±7.1 pmol 10^−5^ cells) is also evidenced after SA exposure. At 3 h after incubation with 0.6 mM ALA in the absence of SA, the intracellular ALA is almost completely consumed and 15.1±1.1 pmol PBG 10^−5^ cells and 13.0±0.7 pmol porphyrin 10^−5^ cells are formed.

In Figure 1A

**Figure 1:**
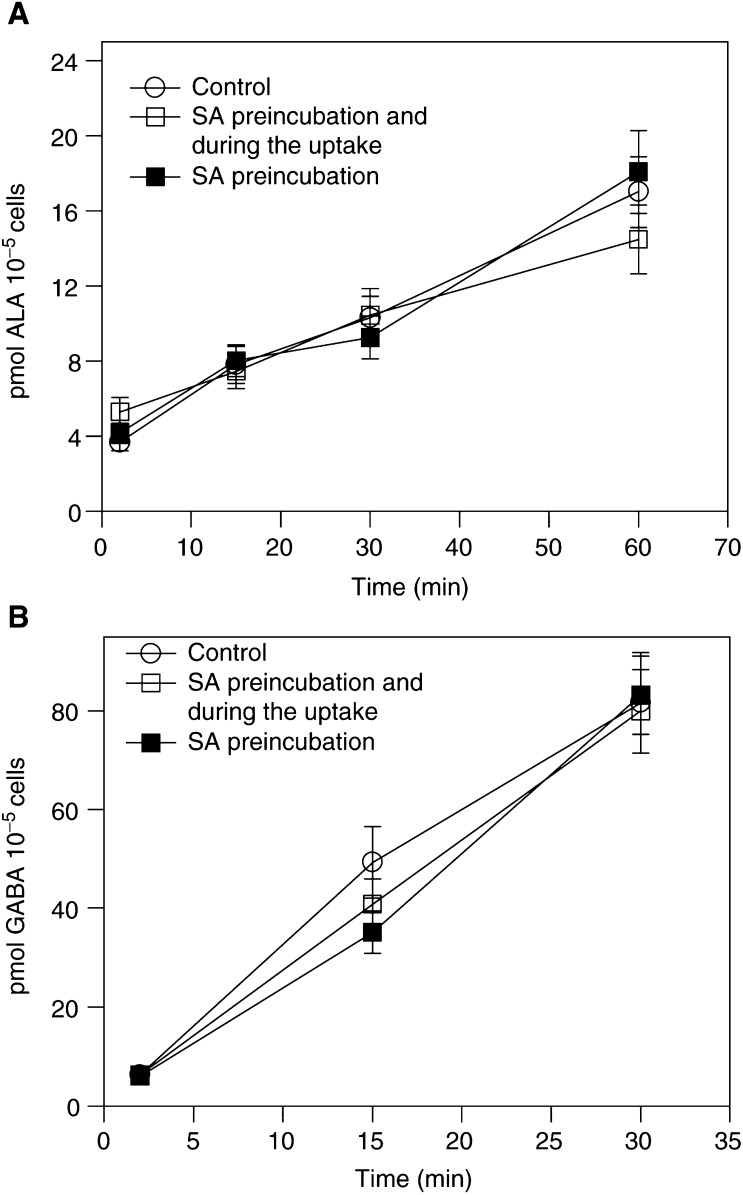
ALA and GABA uptake in the presence of the haem inhibitor SA. Cells were preincubated with 0.5 mM SA and 0.1 mM ^14^C-ALA (**A**) or GABA (**B**) uptake was measured in the presence or absence of SA. Control, classical ALA uptake (*n*=4).

, ALA uptake was measured in cells treated with SA. We choose 0.1 mM ALA for this experiment to minimize ALA conversion (Bermúdez Moretti *et al*, 2002). When the cells were preincubated for 20 min with 0.5 mM SA and this compound was withdrawn before adding ^14^C-ALA, ALA incorporation is similar to that found in control cells. Interestingly, the presence of SA during the whole uptake assay does not modify ALA uptake.

Similarly, Figure 1B shows the effect of SA on GABA transport. Again, there are no changes in GABA incorporation driven by the presence of SA before or during the uptake assay.

In another set of experiments (data not shown), cells treated with 0.5 mM SA or not were preincubated with 0.6 mM ALA for 15, 60 or 180 min before measuring ALA uptake in order to achieve increasing intracellular ALA or porphyrin pools. It was found that initial ALA uptake rates remain constant (0.113±0.015 pmol ALA 10^−5^ cells min^−1^).

In Table 2

**Table 2:** Dependence of ALA and GABA efflux on temperature

	**% of release**	
**Efflux of**	**37°C**	**0°C**
ALA	32	31
GABA	35	18

Cells were preloaded for 15 min with 0.6 mM ^14^C-ALA or GABA, washed with PBS containing either ALA or GABA, respectively, and further incubated for 15 min at 37 or 0°C. ALA or GABA released to the medium was calculated as a percentage of intracellular radioactivity at 0 min after ALA loading (initial intracellular pools).

, we studied the dependence on temperature of ALA and GABA efflux. A 32% efflux of ALA is detected at 37°C and similar results were obtained when it was measured at 0°C. In contrast, GABA efflux decreases significantly to 18% when the temperature is lowered to 0°C.

Table 3

**Table 3:** ALA and porphyrin efflux in the presence of the haem synthesis inhibitor SA

		**60 min**		**180 min**	
		**−SA**	**+SA**	**−SA**	**+SA**
ALA, PBG and porphyrins^a^	Intracellular	12.50±1.31	9.50±1.12	12.09±1.02	11.26±1.08
	Extracellular	6.79±0.54	8.00±0.75	7.43±0.84	10.52±1.19
					
Porphyrins^b^	Intracellular	12.50±0.9	2.60±0.05	12.09±0.7	4.36±0.07
	Extracellular	1.40±0.04	ND	1.11±0.06	ND
					
ALA and PBG^c^	Intracellular	0±2.20	6.90±1.17	0±1.72	6.90±1.15
	Extracellular d	5.39±0.58	8.00±0.75	6.32±0.9	10.52±1.19

Cells were preloaded for 15 min with 0.6 mM ^14^C-ALA, washed with PBS and further incubated for 60 and 180 min at 37°C. Intracellular and extracellular radioactivity was quantified. In addition, porphyrins were quantified spectrophotometrically and expressed per 10^5^ cells. In +SA experiments, 0.5 mM of the haem synthesis inhibitor was present during the preload and efflux incubations.

a All expressed as pmol ^14^C-ALA, calculated as the addition of both intracellular and extracellular radioactivity.

b pmol ALA converted into porphyrins.

c Expressed as pmol ALA calculated subtracting^b^ from^a^, intracellular and extracellular, respectively.

d *P*<0.005. ND: nondetectable by this method.

shows the results of ALA and porphyrin efflux in the presence or absence of SA. After 15 min of preloading with ALA, in the absence of SA, a complete conversion of ALA into porphyrins is already observed at 60 min, and under these conditions, 15% of the intracellular tetrapyrrole accumulation is released to the medium. The same pattern is observed at longer periods (180 min). When porphyrin synthesis is prevented by SA exposure, only basal levels of tetrapyrroles are detected together with an intracellular ALA pool.

We expressed as ALA+PBG, the result of the subtraction of porphyrins from the total radiolabelled content, because at these times, we are in the presence of ALA metabolisation in the case of +ALA+SA. Efflux of ALA/PBG to the medium is significantly higher in the presence of SA both at 60 and 180 min of incubation.

Table 4

**Table 4:** Porphyrin synthesis in cells exposed to ALA and GABA

	**pmol intracellular porphyrin 10^−5^ cells**	**pmol extracellular porphyrin 10^−5^ cells**
−ALA−GABA	0.56±0.02	0.50±0.02
+ALA	2.10±0.03	1.02±0.05
+ALA+GABA	1.72±0.05	0.85±0.09
+ALA → GABA	2.10±0.04	0.84±0.04
+ALA → ALA	9.8±0.08	1.68±0.05

Cells were loaded by 15 min exposure to 0.6 mM ALA in PBS (+ALA); or with ALA and 0.6 mM GABA (+ALA+GABA). Afterwards, the cells were washed four times and further incubated for 3 h in PBS. +ALA → ALA and+ALA → GABA correspond to cells preloaded for 15 min with ALA, but were further exposed for 3 h either to 0.6 mM ALA or GABA, respectively. Porphyrins were extracted and quantified according to the procedures described in Materials and Methods.

shows porphyrin synthesis in cells exposed to ALA and GABA. Intracellular porphyrins as well as porphyrins released to the medium are 20% lower after coincubation with an equimolar GABA concentration (ALA+GABA). When GABA is added after withdrawal of ALA (+ALA → GABA), porphyrin values are not affected, showing that GABA does not modify ALA efflux, at least to the extent of modifying porphyrin synthesis. These results correlate well with an unchanged rate of ^14^C-ALA efflux upon exposure to GABA or ALA after ALA preloading (data not depicted). As expected, further incubation with ALA for 3 h (+ALA → ALA) correlates with an increase in tetrapyrrole formation.

## DISCUSSION

To determine whether intracellular accumulated ALA affects its transport process in murine mammary adenocarcinoma cells, ALA metabolisation was prevented using SA, a specific competitive inhibitor of ALA-dehydratase (Ebert *et al*, 1979). This enzyme catalyses the condensation of two molecules of ALA to form PBG, the pyrrolic precursor of porphyrins. We proved that under our set of conditions (Table 1), SA completely eliminates PBG and porphyrin synthesis from ALA in this adenocarcinoma cell line. The presence of the inhibitor also leads to an increase of the intracellular ALA pool.

After 3 h of ALA exposure, in the absence of SA, 13.0±0.7 pmol porphyrin 10^−5^ cells were synthesised. A linear increase in porphyrin synthesis up to 3 h has been previously established for this cell line (Bermúdez Moretti *et al*, 2002).

As it has already been shown, for 3 h ALA exposure, the incubation with ALA for different times in the presence or absence of SA leads to the intracellular accumulation of ALA or porphyrins, respectively. To determine the effect of different intracellular ALA and porphyrin pools on ALA transport, we treated the cells with or without SA before preincubating with ALA for different times and before measuring ALA uptake (data not shown). We found that neither ALA nor porphyrin pools regulate ALA incorporation, since the initial ALA uptake rates remain constant. In addition, the presence of SA during the whole assay without changing ALA uptake demonstrates that SA does not compete for ALA uptake (Figure 1), in good agreement with the findings of Gibson *et al* (2001).

As it is well known that GABA is a substrate for the BETA transporters (Palacín *et al*, 1998), the effect of SA on GABA transport was also examined and, as expected, there is no competition between GABA and SA, and inhibition of ALA metabolisation did not affect GABA uptake either.

The higher efflux of ALA/PBG in the presence of SA (Table 3) may be a consequence of a high intracellular ALA pool due to an impairment on the conversion of ALA into PBG, which in addition, might be less diffusible than ALA.

According to temperature dependence data (Table 2), ALA transport from the cytoplasm to the extracellular medium is carried out by a diffusion process, at least after this short incubation time period. On the other hand, only active transport seems to be involved in GABA efflux. These were the expected results since they are in agreement with our previous findings (Bermúdez Moretti *et al*, 2002), where we demonstrated that ALA is incorporated into these cells through two different mechanisms, diffusion and an active process, while GABA uptake is not mediated by diffusion.

When we expose cells to 0.6 mM ALA for 15 min, we can see that after 60 min of ALA withdrawal, all the ALA has been consumed and porphyrin synthesis cannot be further increased by extending the incubation time to 180 min (Table 3). Some haem enzymes such as ALA dehydratase and PBG deaminase have been postulated to be rate limiting in the production of porphyrins from ALA (Gibson *et al*, 1998,^2001^). However, under our conditions of short ALA exposure, ALA availability is limiting tetrapyrrole formation.

GABA is a competitor for ALA uptake and a putative competitor for ALA efflux. In an attempt to diminish ALA efflux, and consequently, increase porphyrin synthesis, we exposed the cells to equimolar GABA concentrations, after ALA preloading (Table 4). However, neither ALA nor GABA affected ALA efflux, instead the latter competed for ALA uptake. These data reinforce the hypothesis that ALA efflux is mainly mediated by a diffusion process, whereas ALA uptake even at short incubation times, is at least in part mediated by a saturable transport mechanism.

Porphyrin release is kept to 15% independent of the incubation times. This is a typical release of PPIX to a medium without serum, and it has been previously reported by Fukuda *et al* (1993b) and Iinuma *et al* (1994). Although it is not known whether this loss is passive or whether it involves an active transport process (Fukuda *et al*, 1993a,1993b), the different efflux rates observed by Iinuma *et al* (1994) using different cell lines, suggest that the mechanism is not simply mediated by diffusion. However, characterisation of the mechanisms of PPIX efflux exceeds the aims of this paper.

This is the first study describing ALA efflux in a non-neural cell line. We attempted to diminish such efflux to improve ALA availability with the aim of increasing PDT efficacy. However, we found that until 15 min after preloading with ALA, a diffusion process mediates efflux, and we could not modulate it by employing putative competitors. Nevertheless, further studies should be carried out to explore the possibility of inhibiting ALA efflux at longer incubation times.

The design of new nondiffusible ALA derivatives would be an approach to overcome the disadvantage of ALA efflux. In this regard, taking into account the fact that GABA does not efflux from cells, the structural differences between ALA and GABA could be further exploited.
